# Determining the Optimal Administration Conditions under Which MIF Exerts Neuroprotective Effects by Inducing BDNF Expression and Inhibiting Apoptosis in an In Vitro Stroke Model

**DOI:** 10.3390/brainsci11020280

**Published:** 2021-02-23

**Authors:** Chul Jung, Mi Hee Kim, Ye Yeong Kim, Ji Ae Kim, Eun Jae Ko, Seung Hak Lee, Dae Yul Kim

**Affiliations:** 1 Asan Medical Center, Department of Rehabilitation Medicine, University of Ulsan College of Medicine, Seoul 05505, Korea; speciron90@gmail.com (C.J.); jirehjiae@gmail.com (J.A.K.); gonjae0610@gmail.com (E.J.K.); seunghak@gmail.com (S.H.L.); 2 Asan Medical Center, Asan Institute for Life Sciences, University of Ulsan College of Medicine, Seoul 05505, Korea; algml7843@naver.com (M.H.K.); dpdud5567@nate.com (Y.Y.K.)

**Keywords:** macrophage migration inhibitory factor, neuroprotection, brain-derived neurotrophic factor, apoptosis, ischemic stroke

## Abstract

Macrophage migration inhibitory factor (MIF) exerts neuroprotective effects against cerebral ischemia/reperfusion injury by inhibiting neuronal apoptosis and inducing the expression of brain-derived neurotrophic factor (BDNF). However, the optimal administration conditions of MIF are currently unknown. Here, we aimed to identify these conditions in an in vitro model. To determine the optimal concentration of MIF, human neuroblastoma cells were assigned to one of seven groups: control, oxygen and glucose deprivation/reperfusion (OGD/R), and OGD/R with different concentrations (1, 10, 30, 60, and 100 ng/mL) of MIF. Six groups were studied to investigate the optimal administration time: control, OGD/R, and OGD/R with MIF administered at different times (pre-OGD, OGD-treat, post-OGD, and whole-processing). Water-soluble tetrazolium salt-1 assay, Western blot analysis, and immunocytochemistry were used to analyze cell viability and protein expression. We found that 60 ng/mL was the optimal concentration of MIF. However, the effects of administration time were not significant; MIF elicited similar neuroprotective effects regardless of administration time. These findings correlated with the expression of BDNF and apoptosis-related proteins. This study provides detailed information on MIF administration, which offers a foundation for future in vivo studies and translation into novel therapeutic strategies for ischemic stroke.

## 1. Introduction

Globally, 100 million people have experienced a stroke, which is the leading cause of long-term disability [1,2,3]. Ischemic stroke is caused by the attenuation of blood supply to the brain and accounts for 80% of all stroke cases. As the global population ages and risk factors accumulate, the prevalence rate of ischemic stroke has increased [1]. Tissue plasminogen activator (tPA) is the only approved pharmacological treatment for ischemic stroke; however, thrombolytic therapy with tPA must be administered to patients within 4.5 h of stroke onset. In Korea, only 11.7% of patients with ischemic stroke have received thrombolytic therapy with tPA [4]; therefore, the translation of novel molecules with brain-protective effects into therapies with high efficiency and a wide therapeutic time window is vital [5,6,7,8,9].

Brain parenchyma injured by ischemic stroke can be divided into the ischemic core and the penumbra. The ischemic core consists of neurons that undergo irreversible necrosis in rapid response to the deprivation of nutrient supply. In contrast, the neurons of the penumbra are mildly injured, and their damage may be reversible [5]. The activation of apoptotic pathways and inflammatory responses subsequently occurs in ischemic regions, which injure the remaining neurons in the penumbra [10,11]. Novel therapeutic strategies have focused on inhibiting apoptosis and modulating inflammatory responses. These may lead to the protection of mildly injured neurons in the penumbra [5,11].

Macrophage migration inhibitory factor (MIF) is a cytokine expressed in various types of cells, including neurons, non-neuronal cells, and neural progenitors. It is secreted in response to hypoxia [12,13]. Secreted MIF binds to several receptors and exerts its biological function of inhibiting apoptosis and promoting inflammation [12]. The role of MIF has been investigated in various neurological diseases. MIF is detrimental to brain tumors, Alzheimer’s disease, and spinal cord injury and protective in amyotrophic lateral sclerosis [14,15]. Interestingly, MIF has been reported to have both protective and detrimental effects on ischemic stroke. Previous studies have reported that MIF plays a protective role in ischemic stroke by suppressing apoptosis [13,16,17], whereas others have described MIF as having a detrimental effect by promoting inflammation [18,19,20] or disrupting the tight junction in brain endothelial cells [21].

Our previous studies have reported that MIF mainly plays a neuroprotective role in ischemic stroke [22,23]. In these studies, we found that MIF was neuroprotective in ischemic stroke models via the inhibition of neuronal cell apoptosis and induction of brain-derived neurotrophic factor (BDNF) expression. BDNF is expressed in various brain regions and exerts neuroprotective effects via multiple mechanisms, such as well-established neurotrophic actions that may lead to neurogenesis, and anti-apoptosis and anti-oxidation [24,25]. The induction of BDNF expression has also been suggested as a mechanism underlying the therapeutic effects of MIF on depression [26]. However, the administration conditions for optimizing the neuroprotective effects of MIF are currently unclear.

The aim of this study was to investigate the neuroprotective effects of MIF under different concentration and time of administration conditions. Additionally, we sought to identify the optimal administration conditions under which MIF exerted the best neuroprotective effects in an in vitro stroke model. The neuroprotection provided by MIF was explored according to its effects on BDNF expression and apoptosis, separately.

## 2. Materials and Methods

### 2.1. Cell Cultures

The human neuroblastoma cell line, SH-SY5Y (American Type Culture Collection, Manassas, VA, USA), was used in this study. As a commercialized cell line was used, ethics committee approval was waived. Differentiated cells from passages of 26 to 30 were used and cultured in Dulbecco’s modified Eagle’s medium (DMEM, Gibco, Carlsbad, CA, USA) supplemented with 10% fetal bovine serum (Biowest, Nuaillè, France), and 100 µg/mL streptomycin and 100 U/mL penicillin (Pen strep, Gibco) and incubated in a humidified atmosphere containing 95% air/5% CO_2_ at 37 °C.

### 2.2. Oxygen and Glucose Deprivation/Reperfusion (OGD/R)

The OGD/R in vitro model mimicking cerebral ischemia/reperfusion (I/R) injury was established as previously described [21]. To establish OGD cultures, cell media were exchanged with glucose-free DMEM with the dissolved oxygen removed, and cultures were then transferred into an incubator containing multi-gas mixture with 1% O_2_ at 37 °C for 4 h. During reperfusion, cell media were replaced with normal media and cultures were reoxygenated in an incubator under 95% air/5% CO_2_ at 37 °C for 24 h.

### 2.3. MIF Administration

**Figure 1:**
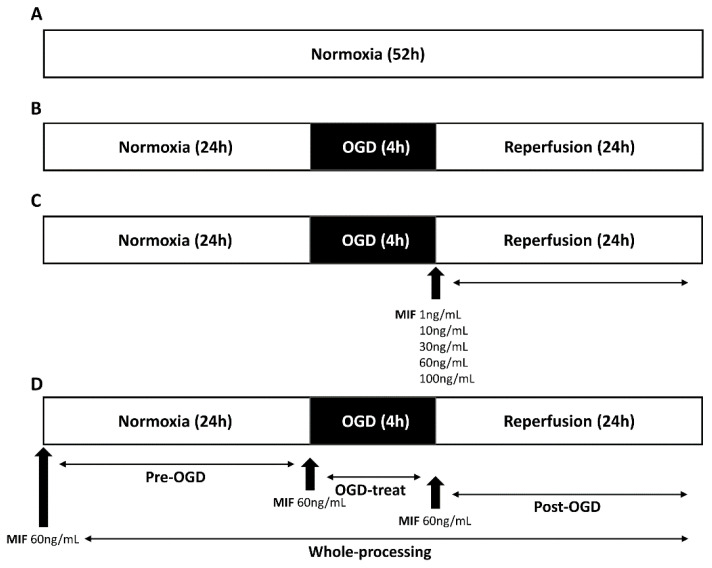
Schematic diagram of the study protocol. (**A**) Control group; (**B**) oxygen and glucose deprivation/reperfusion (OGD/R) group; (**C**) OGD/R group treated with different concentrations of macrophage migration inhibitory factor (MIF); (**D**) OGD/R group treated with MIF administered at different times.

Figure 1 shows a schematic diagram of the study protocol. All the cells were simultaneously fixed at the endpoint of 24 h of reperfusion and then treated as samples.

#### 2.3.1. MIF Administration at Different Concentrations

**Figure 2:**
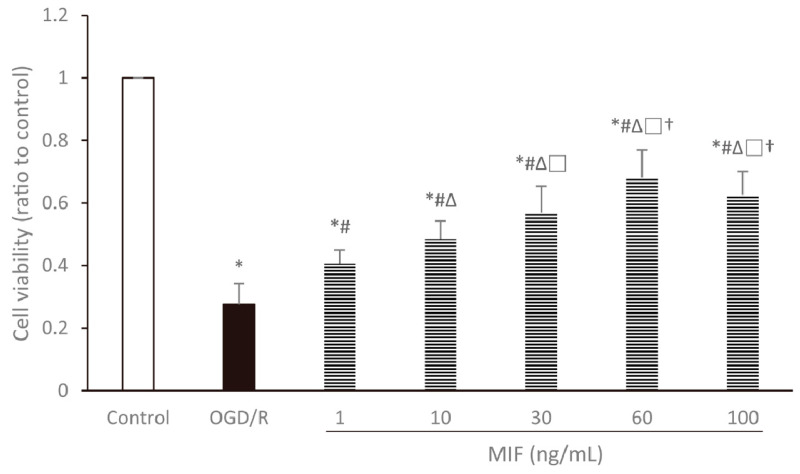
Cell viability assessed by water-soluble tetrazolium salt-1 (WST-1) assay relative to the control group. Cell viability was significantly increased in the MIF groups compared with the OGD/R group and peaked in the 60 ng/mL MIF group. Mean ± SD values from 24 independent experiments are presented. * *p* < 0.05 vs. control; # *p* < 0.05 vs. OGD/R group; ∆ *p* < 0.05 vs. 1 ng/mL MIF group; □ *p* < 0.05 vs. 10 ng/mL MIF group; † *p* < 0.05 vs. 30 ng/mL MIF group. Differences between groups were analyzed using one-way ANOVA followed by Tukey’s post hoc test.

To determine the optimal MIF concentration to administer, we studied the following seven groups: (1) a control group, in which the cells were maintained under normoxic conditions for 52 h (Figure 1A); (2) an OGD/R group, in which the cells under normoxic conditions for 24 h were subjected to 4 h of OGD, followed by 24 h of reperfusion (Figure 1B); and (3–7) MIF treatment groups, wherein the cells under normoxic conditions for 24 h were subjected to 4 h of OGD, followed by treatment with different concentrations (1, 10, 30, 60, and 100 ng/mL) of recombinant MIF (Abcam, Cambridge, UK) during 24 h of reperfusion (Figure 1C).

#### 2.3.2. MIF Administration at Different Times

**Figure 3:**
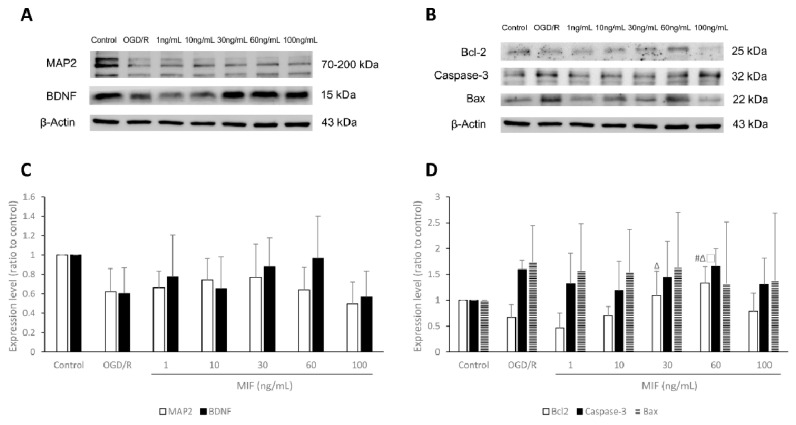
Western blot analysis of microtubule associated protein 2 (MAP2), brain-derived neurotrophic factor (BDNF), B-cell lymphoma 2 (Bcl-2), Caspase-3, and Bcl-2-associated X (Bax) expression under the control, OGD/R, and OGD/R conditions treated with different concentrations of MIF. (**A**,**B**) Representative bands of each group in the Western blot; (**C**,**D**) the expression of Bcl-2 was significantly higher in the 60 ng/mL MIF group than in the OGD/R group. Mean ± SD values are presented. # *p* < 0.05 vs. OGD/R group; ∆ *p* < 0.05 vs. 1 ng/mL MIF group; □ *p* < 0.05 vs. 10 ng/mL MIF group. Differences between the groups were analyzed using one-way ANOVA followed by Tukey’s post hoc test (number of independent cell culture experiments (*n*): all, *n* = 5).

To investigate the optimal time for MIF administration, we studied the following six groups: (1) a control group, in which the cells were maintained under normoxic conditions for 52 h (Figure 1A); (2) an OGD/R group, in which the cells under normoxic conditions for 24 h were subjected to 4 h of OGD, followed by 24 h of reperfusion (Figure 1B); (3) a pre-OGD group in which the cells were treated with MIF for 24 h before OGD, followed by 4 h of OGD and 24 h of reperfusion; (4) an OGD-treat group, in which the cells under normoxic conditions for 24 h were treated with MIF during 4 h of OGD, followed by 24 h of reperfusion; (5) a post-OGD group, in which the cells under normoxic conditions for 24 h were subjected to 4 h of OGD, followed by MIF treatment during 24 h of reperfusion; and (6) a whole-processing (WP) group, in which the cells were treated with MIF 24 h before and during 4 h of OGD administration and 24 h of reperfusion. The cells were administered a concentration of 60 ng/mL recombinant MIF, which had shown the best neuroprotective effect (Figure 1D). 

### 2.4. Water-Soluble Tetrazolium Salt-1 (WST-1) Assay

Cell viability was evaluated using the WST-1 assay. Human neuroblastoma cells were seeded onto 96-well plates (2 × 10^4^ cells/well) and subjected to various treatments as described above. The cell media were carefully aspirated, and cells were washed with serum-free DMEM. Then, 10 µL of WST-1 (Roche, Basel, Switzerland) was added to each culture well and filled with 100 µL of serum-free DMEM. Next, cell cultures were incubated for 1.5 h at 37 °C. Cell survival rates were measured by analyzing the absorbance at 440 nm for each well using a microplate reader (Tecan, Männedorf, Switzerland). Results were expressed as percentages of viable cells compared with the value of the control.

### 2.5. Western Blot Analysis

We analyzed the protein expression of microtubule associated protein 2 (MAP2) and BDNF, and B-cell lymphoma 2 (Bcl-2), Caspase-3, and Bcl-2-associated X (Bax) to compare BDNF expression and apoptotic activity among the groups.

Cell pellets were collected via centrifugation and the collected cells were washed. The addition of RIPA buffer (200 µL/well, Thermo Fisher Scientific, Waltham, MA, USA) lysed the cells, and total proteins were extracted from the cell lysates. A Bradford assay was used to quantify the amount of extracted proteins, and 1 µg/µL of sample was prepared. The samples were diluted in 5× sodium dodecyl sulfate-poly-acrylamide gel electrophoresis (SDS-PAGE) sample loading buffer and heated at 95 °C for 7 min. Proteins were electrophoresed using SDS-PAGE and electro-transferred onto a nitrocellulose membrane. After blocking for 1 h with 5% nonfat dry milk in Tris-buffered saline with Tween (TWEEN 20, Sigma, St. Louis, MO, USA; TBS-T), the membranes were incubated with primary antibodies overnight at 4 °C. The following primary antibodies were used: anti-MAP2 (1:1000; Thermo Fisher Scientific), anti-BDNF (1:1000; Abcam), anti-Bcl-2 (1:1000; Abcam), anti-Caspase-3 (1:1000; Abcam), and anti-Bax (1:1000; Abcam). After washing twice in TBS-T, the membranes were incubated with horseradish-peroxidase-conjugated secondary antibodies (1:5000; anti-mouse IgG antibodies, GeneTex, Irvine, CA, USA; anti-rabbit IgG antibodies, Enzo Life Science, New York, NY, USA) for 1 h. After washing another three times in TBS-T, the protein bands were visualized using enhanced chemiluminescence detection reagents (Thermo Fisher Scientific). The results were quantified using Image Studio Lite ver. 5.2 (LI-COR Inc., Lincoln, NE, USA). All results were normalized to the levels of β-actin, which was used as a loading control. The relative amount of immunoreactivity to the control group was expressed.

### 2.6. Immunocytochemistry

We analyzed the expression of MAP2, BDNF, Bcl-2, Caspase-3, and Bax proteins with immunocytochemistry to compare cellular responses among the groups.

Cells (1 × 10^5^ cells/well) were seeded onto a ReproCoat (Reprocell, Beltsville, MD, USA) pre-coated cover slip and fixed with 2% paraformaldehyde (PFA) at room temperature for 10 min. The cells were re-fixed with 4% PFA for 7 min and washed twice (5 min each) in phosphate-buffered saline (PBS). The cells were permeated with 0.1% Triton X-100 (Sigma) for 10 min and washed again. Next, the cells were blocked with 5% normal goat serum (Thermo Fisher Scientific)/5% BSA/0.1% tPBS (triton-PBS) for 1 h and incubated overnight at 4 °C with primary antibodies diluted in 5% BSA/0.1% tPBS. The following primary antibodies were used: anti-MAP2 (Thermo Fisher Scientific), anti-BDNF (Abcam), anti-Bcl-2 (Abcam), anti-Caspase-3 (Abcam), and anti-Bax (Abcam). After washing twice (5 min each) in PBS, the cells were incubated with secondary antibodies (1:1000; Alexa Fluor 488 conjugated donkey anti-rabbit IgG antibodies, Abcam; Alexa Fluor 568 conjugated goat anti-mouse IgG antibodies, Abcam) at room temperature for 90 min. After washing twice in PBS (5 min each), the cell nuclei were stained with 10 µL of 4′,6-diamidino-2-phenylindole (DAPI, Invitrogen, Carlsbad, CA, USA) overnight. Images were captured using confocal microscopy (confocal laser scanning microscope; LSM 880, Carl Zeiss, Jena, Germany), and the results were quantified using Image J ver. 1.48 (Carl Zeiss). The fluorescence intensity was divided by DAPI intensity to represent the intensity per living cell.

### 2.7. Statistical Analysis

All statistical analyses were performed using SPSS for Windows version 25 (IBM SPSS Inc., Chicago, IL, USA). Differences were considered statistically significant if *p* < 0.05. Data are presented as mean ± standard deviation (SD). One-way analysis of variance (ANOVA) and Tukey’s post hoc test were used to analyze between group differences. Each experiment was repeated at least five times.

## 3. Results

### 3.1. Optimal Concentration of MIF Administration

#### 3.1.1. Cell Viability Is the Highest in the 60 ng/mL MIF Group

Cell viability under conditions of normoxia, OGD/R, and OGD/R treated with different concentrations of MIF is compared in Figure 2. The results of the WST-1 assay performed after OGD/R injury showed that cell survival rates significantly decreased in the OGD/R and MIF groups when compared with that in the control group (*p* < 0.05). Compared with the OGD/R group, cell survival rates were higher in the MIF groups and showed a concentration-dependent increase (*p* < 0.05). The highest cell viability was observed in the 60 ng/mL MIF group; therefore, we selected this concentration for further experiments assessing the optimal MIF administration time. 

As there were no significant differences in the viability of the cells treated with 60 and 100 ng/mL MIF, additional experiments were performed to investigate the cell viability under OGD/R and treated with higher concentrations (e.g., 200 and 1000 ng/mL) of MIF. These experiments also revealed that the 60 ng/mL MIF resulted in the highest cell viability; all data can be viewed in Supplementary Figure S1.

#### 3.1.2. Bcl-2 Expression Is Significantly Increased in the 60 ng/mL MIF Group

Figure 3 shows comparisons between the expression of MAP2, BDNF, Bcl-2, Caspase-3, and Bax proteins. Western blot analysis after OGD/R injury showed that the expression of Bcl-2, an anti-apoptosis protein, significantly increased in the 60 ng/mL MIF group when compared with that in the OGD/R group (Figure 3B,D; *p* < 0.05). Although it was not statistically significant, the expression of MAP2 and BDNF proteins increased in most MIF groups when compared with that in the OGD/R group (Figure 3A,C).

#### 3.1.3. Inducing BDNF Expression and Inhibiting Apoptosis Is Most Effective in the 60 ng/mL MIF Group

**Figure 4:**
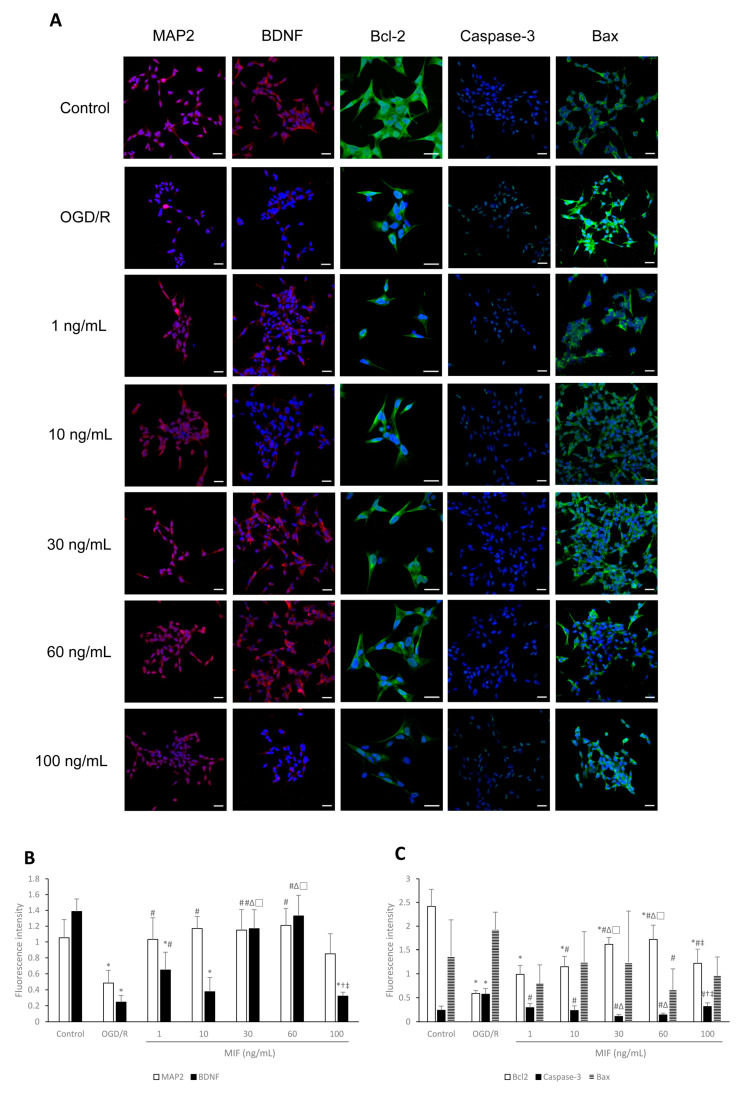
Immunocytochemistry findings of MAP2, BDNF, Bcl-2, Caspase-3, and Bax expression levels under the conditions of control, OGD/R, and OGD/R treated with different concentrations of MIF. (**A**) Representative images of immunocytochemistry for each group; (**B**,**C**) the expression of MAP2, BDNF, and Bcl-2 was significantly higher in the 60 ng/mL MIF group than in the OGD/R group. The expression of Caspase-3 and Bax was significantly lower in the 60 ng/mL MIF group than in the OGD/R group. Mean ± SD values are presented. * *p* < 0.05 vs. control; # *p* < 0.05 vs. OGD/R group; ∆ *p* < 0.05 vs. 1 ng/mL MIF group; □ *p* < 0.05 vs. 10 ng/mL MIF group; † *p* < 0.05 vs. 30 ng/mL MIF group; ‡ *p* < 0.05 vs. 60 ng/mL MIF group. The differences among the groups were analyzed using one-way ANOVA followed by Tukey’s post hoc test (number of independent cell culture experiments (*n*): all, *n* = 6) (scale bar, 20 µm; magnification, 400× for MAP2, BDNF, Caspase-3, and Bax, or 630× for Bcl-2).

The expression of MAP2, BDNF, Bcl-2, Caspase-3, and Bax proteins per viable cell is presented and compared in Figure 4. After OGD/R injury, immunocytochemistry results showed that the expression of MAP2 and BDNF proteins significantly increased in most MIF groups when compared with the OGD/R group (Figure 4A,B; *p* < 0.05). BDNF expression was significantly higher in the 1, 30, and 60 ng/mL MIF groups than in the OGD/R group, and this was highest in the 30 and 60 ng/mL MIF groups (Figure 4A,B; *p* < 0.05). Apoptosis marker expression presented similar trends; Bcl-2 expression was significantly higher in the 10, 30, and 60 ng/mL MIF groups than in the OGD/R group, and highest in the 60 ng/mL MIF group (Figure 4A,C; *p* < 0.05). The expression of Caspase-3, an apoptosis indicator, significantly decreased in the MIF groups when compared with the OGD/R group and was the lowest in the 30 and 60 ng/mL MIF groups (Figure 4A,C; *p* < 0.05). The expression of Bax, another apoptosis protein, was significantly lower in the 60 ng/mL MIF group than in the OGD/R group (Figure 4A,C; *p* < 0.05).

These results suggest that 60 ng/mL of MIF, previously set as the optimal concentration, most effectively induced BDNF expression and inhibited the apoptosis of neuroblastoma cells after OGD/R injury in vitro.

### 3.2. Optimal Time of MIF Administration

#### 3.2.1. Cell Viability Is Increased at a Similar Degree in the MIF Groups Regardless of Administration Time

**Figure 5:**
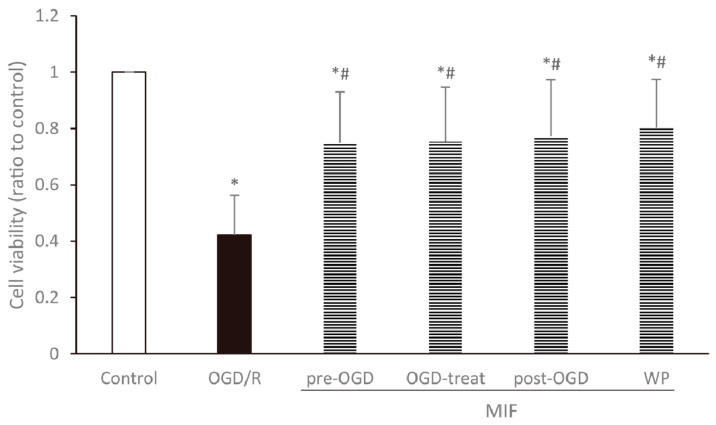
Cell viability assessed using the WST-1 assay relative to the control group. Cell viability significantly increased in the MIF groups compared with the OGD/R group. Mean ± SD values from 30 independent experiments are presented. * *p* < 0.05 vs. control; # *p* < 0.05 vs. OGD/R group. The differences among the groups were analyzed using one-way ANOVA followed by Tukey’s post hoc test.

Cell viability under conditions of normoxia, OGD/R, and OGD/R with MIF administration at different times is compared in Figure 5. After OGD/R injury, the WST-1 assay showed that cell survival rates in MIF groups were significantly higher than those in the OGD/R group and lower than those in the control group (*p* < 0.05). Cell viability was similar between the MIF groups, with no significant differences.

#### 3.2.2. MAP2 Expression Is Significantly Increased in the OGD-Treat Group

**Figure 6:**
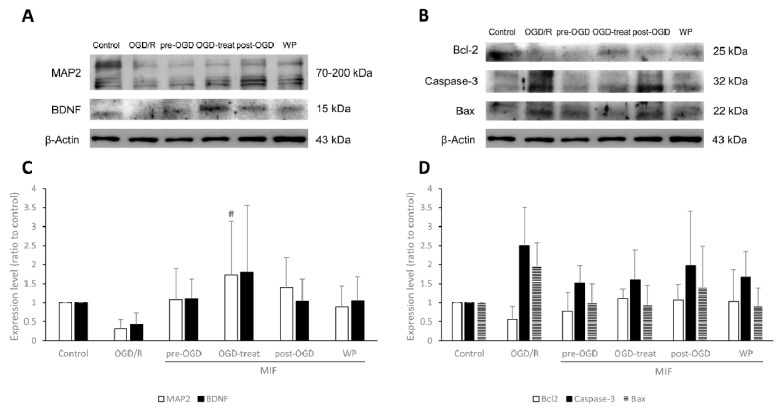
Western blot analysis of MAP2, BDNF, Bcl-2, Caspase-3, and Bax expression under control, OGD/R, and OGD/R conditions administered with MIF at different times. (**A**,**B**) Representative bands of each group in the Western blot; (**C**,**D**) the expression level of MAP2 was significantly higher in the OGD-treat group than in the OGD/R group. Mean ± SD values are presented. # *p* < 0.05 vs. OGD/R group. The differences among the groups were analyzed using one-way ANOVA followed by Tukey’s post hoc test (number of independent cell culture experiments (*n*): MAP2, *n* = 7; BDNF, *n* = 5; Bcl-2, *n* = 5; Caspase-3, *n* = 6; Bax, *n* = 5).

The expression of five proteins (MAP2, BDNF, Bcl-2, Caspase-3, and Bax) was studied in six groups. Figure 6 shows the comparisons of expression between groups. Western blot analysis showed that MAP2 expression was significantly higher in the OGD-treat group than in the OGD/R group (Figure 6A,C; *p* < 0.05). Despite no statistical significance, Bcl-2 expression increased in the MIF groups compared with the OGD/R group; Caspase-3 and Bax expression were lower in the MIF groups than in the OGD/R group (Figure 6B,D).

#### 3.2.3. Inhibiting Apoptosis Is Effective at a Similar Degree in the MIF Groups Regardless of Administration Time; However, BDNF Expression Is Significantly Increased in the Post-OGD Group

**Figure 7:**
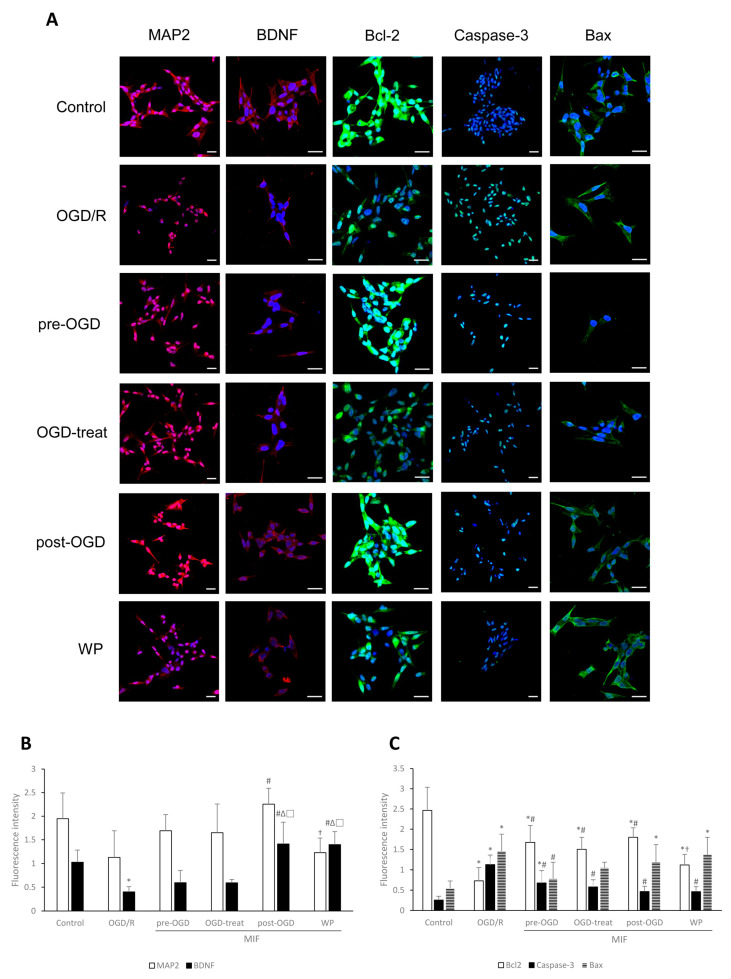
Immunocytochemistry findings of MAP2, BDNF, Bcl-2, Caspase-3, and Bax levels under control, OGD/R, and OGD/R conditions administered with MIF at different times. (**A**) Representative images of immunocytochemistry for each group; (**B**,**C**) the expression of MAP2 and BDNF were higher in the post-OGD group than in the OGD/R group. The expression of Bcl-2 was higher in the MIF groups than in the OGD/R group, and Caspase-3 and Bax expression was lower in the MIF groups than in the OGD/R group. Mean ± SD values are presented. * *p* < 0.05 vs. control; # *p* < 0.05 vs. OGD/R group; ∆ *p* < 0.05 vs. pre-OGD group; □ *p* < 0.05 vs. OGD-treat group; † *p* < 0.05 vs. WP group. Differences between groups were analyzed using one-way ANOVA followed by Tukey’s post hoc test (number of independent cell culture experiments (*n*): all, *n* = 6) (scale bar, 20 µm; magnification, 400× for MAP2 and Caspase-3, or 630× for BDNF, Bcl-2, and Bax).

Figure 7 shows comparisons of the expression of MAP2, BDNF, Bcl-2, Caspase-3, and Bax proteins per living cell. Immunocytochemistry after OGD/R injury showed that MAP2 significantly increased in the post-OGD group compared with that in the OGD/R group (Figure 7A,B; *p* < 0.05). BDNF expression was significantly higher in the post-OGD and WP groups than in the OGD/R, pre-OGD, and OGD-treat groups (Figure 7A,B; *p* < 0.05). However, a slightly different tendency was observed in proteins associated with apoptosis. Bcl-2 expression was significantly higher in the MIF groups, except the WP group, than in the OGD/R group (Figure 7A,C; *p* < 0.05). Caspase-3 expression was significantly decreased in the MIF groups compared with the OGD/R group (Figure 7A,C; *p* < 0.05). Additionally, Bax expression in the pre-OGD group was significantly lower than in the OGD/R group (Figure 7A,C; *p* < 0.05).

These results indicate that MIF administered during reperfusion (post-OGD group) is the most effective at inducing BDNF expression; however, the effects of administration time on inhibiting neuronal apoptosis after OGD/R injury were not significant. The cumulative effects of administration time on cell survival rates did not appear to be critical.

## 4. Discussion

In this in vitro study, we identified the optimal conditions for MIF administration to effectively protect neurons from OGD/R injury. Our optimal concentration experiments revealed that the highest neuronal viability was following 60 ng/mL MIF. This correlated with our data showing that 60 ng/mL MIF was most effective at inducing BDNF expression and inhibiting neuronal apoptosis. However, the effects of administration time appeared to not be critical; different MIF groups showed similar neuroprotective effects. Additionally, the differences in cell viability between MIF groups were not statistically significant. This result may explain the expression of proteins associated with apoptosis in different MIF groups. Further, BDNF expression was significantly higher in the post-OGD group, in which MIF was administered during reperfusion. To the best of our knowledge, this is the first study to investigate the neuroprotective effects of MIF administered under different conditions in the stroke model.

After cerebral I/R injury, the occurrence of inflammatory and immunological reactions injures neurons of the penumbra region, which are considered salvageable at first. Specifically, redundant oxidative stress and calcium influx induces neuronal apoptosis, and inflammatory cells infiltrate the penumbra simultaneously [10,11,27,28]. In this brain microenvironment, MIF, a cytokine involved in apoptosis and inflammation, has been explored for its neuroprotective effects. According to previous studies, MIF exerts both beneficial [13,16,17] and detrimental effects [14,18,19,20,21] on the penumbra regions.

The main neuroprotective action of MIF is to inhibit neuronal apoptosis; it binds to multiple receptors, including CD74, CXCR2, CXCR4, and CXCR7, which triggers downstream signaling leading to inhibited apoptosis [12,13,17]. This signaling mainly suppresses c-Jun N-terminal kinase activation, leading to inhibited apoptosis [13,17]. In addition, MIF exerts neuroprotective effects by inducing BDNF [22,23,26], which may be mediated via the CD74–ERK1/2 pathway [26]. Specifically, a pre-pro form of BDNF protein is first synthesized followed by subsequent removal of the pro-region [24]. Secreted mature BDNF then plays a role in synaptic function, maturation, and differentiation in the central nervous system [24,29,30,31]. In addition to these neurotrophic actions, the BDNF has been reported to exert other neuroprotective effects including suppression of autophagy, anti-oxidation, and anti-apoptosis [25]. By contrast, MIF can have a detrimental effect on neurons via its pro-inflammatory action [14,18,19,20,32]: MIF binds to the receptors of inflammatory cells, regulates their proliferation, which then provokes inflammatory responses. Inflammation after cerebral I/R injury has been suggested to have a deleterious effect that disrupts neural cells and the blood–brain barrier (BBB), leading to the amplification of ischemic injury [33]. Furthermore, though the precise mechanism is yet to be confirmed, MIF itself was also reported to be associated with disruption of the tight junction in brain endothelial cells [21]. The detrimental potential of neuroinflammation and disruption of the tight junction might have contributed to different findings from previous studies.

This study found that 60 ng/mL MIF is most effective at inducing BDNF expression and inhibiting apoptosis. Additionally, cell viability was highest after 60 ng/mL MIF administration. A previous study has reported that the median serum MIF concentration in 292 patients with ischemic stroke was 20.6 ng/mL [19]. While BBB penetration should be taken into consideration, 60 ng/mL MIF is approximately equivalent to three times its physiological level after ischemic stroke. A comparable investigation was performed in myocardial I/R injury. The physiological concentration of MIF in patients with myocardial infarction is reported as 1.5–30 ng/mL [34]; thus, the cardioprotective effects of MIF at different concentrations (1, 10, 50, and 1000 ng/mL) were explored. No MIF concentration showed a significant cardioprotective effect compared with the control [35]. Taken together with our data, these results would have implied that MIF has more therapeutic potential in ischemic stroke than in myocardial infarction.

The effect of MIF administration time on apoptosis and neuronal viability was not significant; MIF administration at a sufficient concentration elicited significant and similar neuroprotective effects regardless of administration time. MIF groups also showed anti-apoptotic activities to a similar degree. However, MIF administered during reperfusion (post-OGD) induced significantly higher BDNF expression. The mechanism for this effect remains unknown; however, this may be supported by the results of a recent study that found that chronic hypoxia exposure reduces the conversion of pro-BDNF into mature BDNF [36]. The expression of mature BDNF would have decreased during OGD, which may explain the obtained results. Based on our previous report identifying that pro-BDNF and mature BDNF shows similar trends following MIF administration, this study only measured mature BDNF expression [23]. Furthermore, these results suggest that MIF is neuroprotective in ischemic stroke mainly via the inhibition of apoptosis rather than through BDNF-associated mechanisms. In line with this, administration time was not critical in the previous myocardial I/R injury study [35]. In the clinical setting, the therapeutic application of MIF can be expected not only in patients with acute ischemic stroke but also in those who are healthy. However, it is difficult to assume the neuroprotective effects of MIF in patients with subacute and chronic ischemic stroke based on this study. Further studies addressing the effects of delayed administration of MIF may lead to expansion of the therapeutic potential of MIF. In addition, these results should be cautiously approached considering the inflammatory and immunological responses occurring after reperfusion. Further in vivo or clinical studies are required to confirm that MIF administration at different times elicits similar neuroprotective effects before proceeding to the therapeutic use of MIF.

There are several limitations to this study. First, we could not address the detrimental effects of MIF due to the limitations of this study being done in vitro. This study identified the effects of MIF on the neural cells and not on the inflammatory or endothelial cells. Paradoxically, however, we could have focused on the neuroprotective effects of MIF because of the study’s settings. Second, a human neuroblastoma cell line was used in this study instead of neuronal cultures. This cell line has the advantages of a shorter doubling time than neurons and of being one of the most commonly used cell types for the investigation of neuroprotection. However, the use of a neuroblastoma cell line can limit the value of the study as a reference for future in vivo/clinical studies. Cancer cells are generally more resistant than other cells to ischemia; therefore, the responses observed in this study may be different from those of neurons. Third, a mechanism study was not performed in this study. The results of this study could not fully explain the effects of MIF, separately from those of BDNF; MIF and BDNF share some neuroprotective mechanisms including anti-apoptosis. Furthermore, this study could not explain why the optimal concentration of MIF was 60 ng/mL. A further in vivo project with a mechanism study is in progress, and may shed some light on these issues. Fourth, the differences among the groups in the Western blot analysis were not that significant compared with the immunocytochemistry. Considering that the results of Western blot analysis showed similar trends with those of immunocytochemistry, we believe the discrepancy might have stemmed from having an insufficient sample size for the Western blot analysis. A larger number of Western blot samples could have increased the statistical power. Since many previous reports have analyzed the immunocytochemistry findings in a relatively qualitative way (e.g., the percentage of cells positive for each marker), the quantitative analysis of fluorescence intensity used in this study could have contributed to this discrepancy as well. Lastly, we did not investigate all the multifaceted mechanisms of MIF in stroke [14]; it is also associated with the metabolic adaptation of cells (glucose metabolism and anti-oxidation) in the initial phase of ischemia [17,37,38].

## 5. Conclusions

In this study, we found that 60 ng/mL was the optimal concentration of MIF administration. Furthermore, MIF elicited similar neuroprotective effects regardless of the administration time. These findings were correlated with the expression of BDNF and proteins associated with apoptosis. This study provides detailed information regarding MIF administration, which offers a foundation for future in vivo studies and translation into novel therapeutic strategies.

## Data Availability

The data presented in this study are available on request from the corresponding author. The data are not publicly available due to data privacy regulations.

## Supplementary Materials

The following are available online at https://www.mdpi.com/2076-3425/11/2/280/s1, Figure S1. Cell viability under conditions of OGD/R treated with higher concentrations of MIF.
