# Supplementary figures and images for: Determining the Optimal Administration Conditions under Which MIF Exerts Neuroprotective Effects by Inducing BDNF Expression and Inhibiting Apoptosis in an In Vitro Stroke Model
Source: Brain Sci. 2021 Feb 23;11(2):280. doi: 10.3390/brainsci11020280 (PMC7926652; doi:10.3390/brainsci11020280)

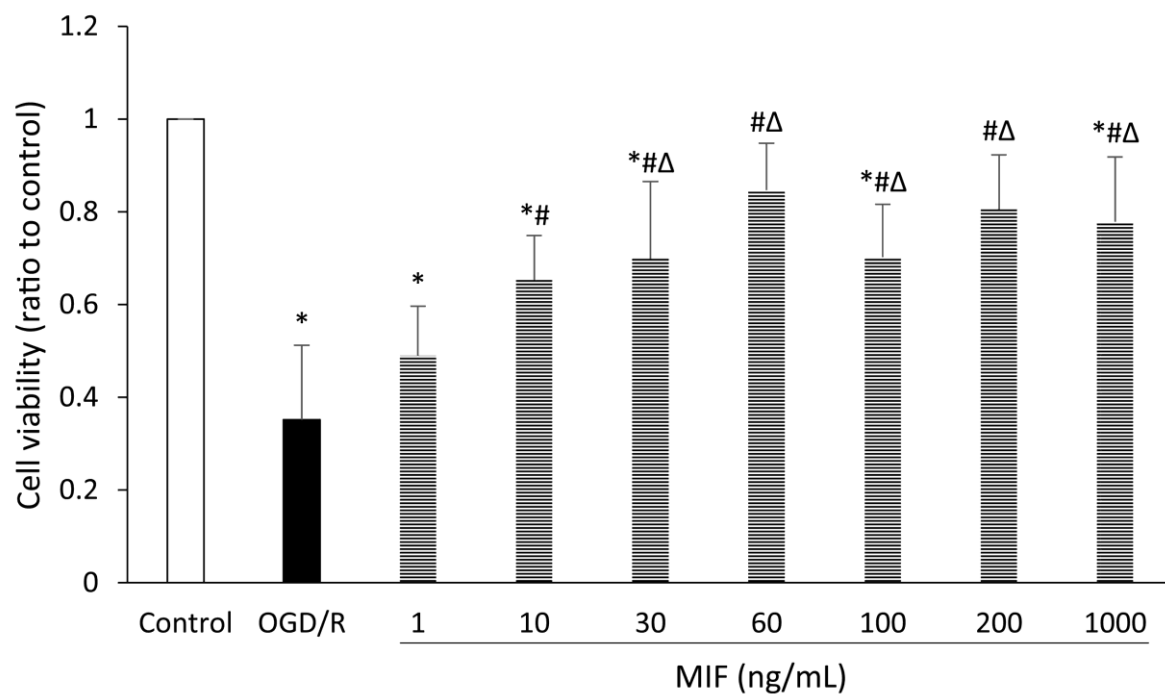

**Figure 1.** Cell viability under conditions of OGD/R treated with higher concentrations of MIF.

Supplement: Supplementary file 1 [file brainsci-11-00280-s001.pdf]
